# The complete mitochondrial genome of *Aleochara postica* Walker, 1858 (Coleoptera: Staphylinidae)

**DOI:** 10.1080/23802359.2021.1938726

**Published:** 2021-06-14

**Authors:** Yanpeng Cai

**Affiliations:** School of Basic Medicine, Guizhou University of Traditional Chinese Medicine, Guizhou, China

**Keywords:** *Aleochara postica*, mitochondrial genome, Staphylinidae, phylogenetic analysis

## Abstract

The complete mitochondrial genome of *Aleochara postica* Walker, 1858 was determined in this study. It is 15,473 bps in length, containing 13 protein-coding genes, 22 tRNA genes, 2 rRNA genes, and a 778 bp A + T-rich control region. Most PCGs use the conventional ATN start codon, except for *cox1* and *nad1*. Two genes (*cox1* and *cox3*) use single T residue as stop codon rather than the routinely used TAA or TAG. All tRNAs, except for TrnS1, exhibit the cloverleaf secondary structure. ML phylogenetic analysis using 13 PCGs of 52 beetle species indicated that *A. postica* was clustered with other members of the subfamily Aleocharinae as conventional taxonomy predicted.

*Aleochara postica* Walker, 1858, a rove beetle species, belongs to the genus *Aleochara* Gravenhorst (Staphylinidae: Aleocharinae: Aleocharini). *Aleochara* with more than 500 species distributed worldwide is not only one of the most speciose genera in Aleocharinae, but also a distinctive group in lifestyle (Klimaszewski 1984; Caron et al. 2019). Most of its species have their larvae specifically feeding on pupae of cyclorrhaphous Diptera as ectoparasitoids, and adult beetles preying on dipteran eggs and larvae (Luo and Zhou 2012; Yamamoto and Maruyama 2016; Caron et al. 2019). Thus, they could be utilized for biological control against noxious flies (Yamamoto and Maruyama 2016; Caron et al. 2019). Nevertheless, no complete mitogenome of *Aleochara* was available so for. In compensation, we present herein the complete mitogenome of *A. postica*, which is widely distributed in China, Japan, Korea, Sri Lanka, and much of Oriental region (Yamamoto and Maruyama 2016). In our study, the adults were collected in 2020, from Guiyang Huaxi District (26°23'06″N, 106°36'56″E, 1163 m), Guizhou, China, using maggoty dead fish bait.

The high-throughput sequencing was performed at Sangon Biotech (Shanghai) Co., Ltd., China, using Illumina HiSeq2500 platform (Illumina, San Diego, CA). The de novo assembly of mitogenome and correctness check were carried out with the software combination of SPAdes V.3.14.1 (Bankevich et al. 2012), MitoZ V.2.3 (Meng et al. 2019), and Pilon V.1.23 (Walker et al. 2014). MITOS Web Server (http://mitos2.bioinf.uni-leipzig.de/index.py) was utilized for annotation. The remaining alcohol-preserved specimen tissue and the total DNA after sequencing were deposited under −20 °C in the Insect Collection of Guizhou University of Traditional Chinese Medicine, Guiyang, China (Yanpeng Cai, cyp815@hotmail.com, Voucher specimens: GZUTCM:002).

The complete circular mitogenome of *A. postica* (GenBank: MW284907) is 15,473 bps in length, containing the typical metazoan mitochondrial genes (13 protein-coding genes, 22 tRNA genes, 2 rRNA genes) and a 778 bp long A + T-rich control region. Most PCGs of *A. postica* use conventional start codons (ATN) and stop codons (TAA or TAG). Whereas, *cox1* and *nad1* genes initiate with putative start codons GAT and TTG respectively, *cox1* and *cox3* genes use single T as incomplete stop codon. Twenty-one out of 22 tRNAs exhibit the typical clover-leaf structure. TrnS1 as the only exception lacks the DHU arm, and that the anticodon of trnS1 is UCU instead of routinely used GCU.

The ML phylogenetic tree was reconstructed using IQTREE V.2.07 (Nguyen et al. 2015) for family Staphylinidae, based on 13 PCGs of *A. postica* plus 51 species obtained from GenBank, among which two representatives of Leiodidae were selected as outgroups (Lin et al. 2018). The partitioning scheme for the three codon positions of the 13 genes was determined by the TESTMERGE option in IQTREE. Ten partitions were finally divided and allocated with their own best fit substitution model and parameters (GTR + F + I + G4, GTR + F + I + G4, GTR + F + I + G4, TN + F+G4, GTR + F + I + G4, TVM + F + I + G4, TN + F+G4, GTR + F + I + G4, K3Pu + F + I + G4, TN + F + I + G4). 1000 replicates of standard bootstrap analysis were executed to produce the bootstrap support values. As a result, seven subfamilies with multiple available representatives (Aleocharinae, Omaliinae, Paederinae, Pselaphinae, Scaphidiinae, Staphylininae, Steninae) were recovered as monophyla. Paederinae was a sibling group to Staphylininae which was supported by multiple previous studies (e.g. Mckenna et al. 2015; Tihelka et al. 2020). Tachyporinae was polyphyletic, which was proved in Yamamoto (2021) as well. The only representative of Habrocerinae was oddly nested in Peaderinae. *Aleochara postica* was clustered in Aleocharinae as morphological taxonomy predicted (Figure 1).

**Figure 1.**
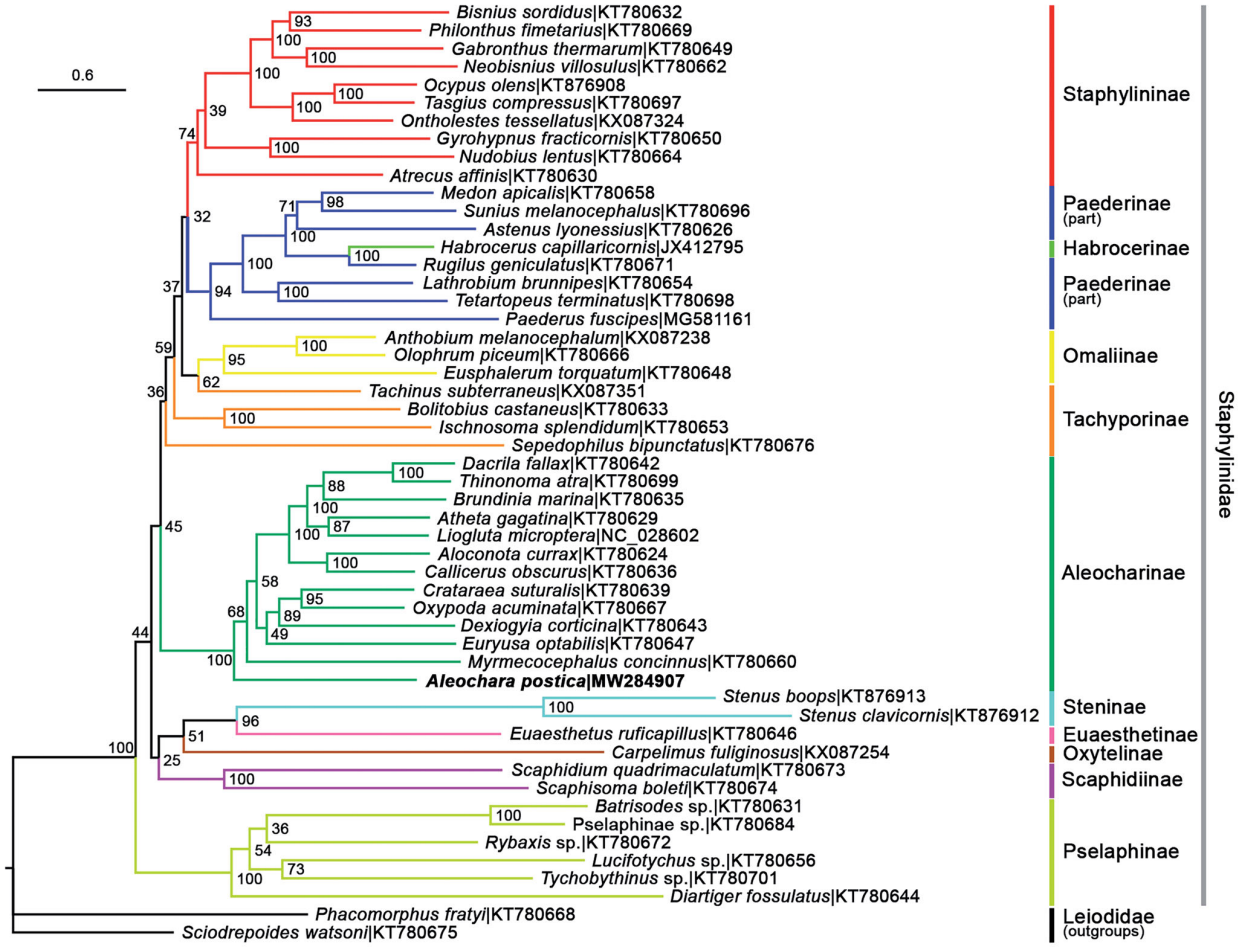
The ML phylogenetic tree was built from *A. postica* (in bold font) and 51 other species. Bootstrap support values were labeled at nodes. GenBank accession numbers of each species were listed in the tree.

## Data Availability

The genome sequence data that support the findings of this study are openly available in GenBank of NCBI at https://www.ncbi.nlm.nih.gov/nuccore/MW284907 under the Accession no. MW284907. The associated BioProject, SRA, and Bio-Sample numbers are PRJNA728529, SRR14508658, and SAMN19091164, respectively.
